# Potential Gains in Reproductive-Aged Life Expectancy by Eliminating Maternal Mortality: A Demographic Bonus of Achieving MDG 5

**DOI:** 10.1371/journal.pone.0086694

**Published:** 2014-02-13

**Authors:** Vladimir Canudas-Romo, Li Liu, Linnea Zimmerman, Saifuddin Ahmed, Amy Tsui

**Affiliations:** 1 Max Planck Odense Center, University of Southern Denmark, Denmark; 2 Department of Population, Family and Reproductive Health, Johns Hopkins Bloomberg School of Public Health, Baltimore, Maryland, United States of America; 3 Department of International Health, Johns Hopkins Bloomberg School of Public Health, Baltimore, Maryland, United States of America; London School of Economics, United Kingdom

## Abstract

**Objective:**

We assessed the change over time in the contribution of maternal mortality to a life expectancy calculated between ages 15 and 49, or Reproductive-Aged Life Expectancy (RALE). Our goal was to estimate the increase in RALE in developed countries over the twentieth century and the hypothetical gains in African countries today by eliminating maternal mortality.

**Methods:**

Analogous to life expectancy, RALE is calculated from a life table of ages 15 to 49. Specifically, RALE is the average number of years that women at age 15 would be expected to live between 15 and 49 with current mortality rates. Associated single decrement life tables of causes of death other than maternal mortality are explored to assess the possible gains in RALE by reducing or eliminating maternal mortality. We used population-based data from the Human Mortality Database and the Demographic and Health Surveys.

**Findings:**

In developed countries, five years in RALE were gained over the twentieth century, of which approximately 10%, or half a year, was attributable to reductions in maternal mortality. In sub-Saharan African countries, the possible achievable gains fluctuate between 0.24 and 1.47 years, or 6% and 44% of potential gains in RALE.

**Conclusions:**

Maternal mortality is a rare event, yet it is still a very important component of RALE. Averting the burden of maternal deaths could return a significant increase in the most productive ages of human life.

## Introduction

In the mid-2000's, for the first time in recorded human history, women in every country gained a longer life expectancy at birth than men [1]. Simultaneously, during the early half of the twentieth century, women's risk of death from pregnancy-related complications reduced by more than 90% in many developed country settings [2]. However, the contribution of maternal mortality reduction on improving women's life expectancy, especially to reproductive age period (15 to 49 years of age) is not well known. Understanding the impact of maternal mortality reduction on life expectancy is particularly relevant in developing countries, where 99% of worldwide maternal deaths occur [3], [4]. With new funding commitments being made and a global momentum being built in support of lowering maternal mortality, we are racing to achieve the Millennium Development Goal 5 to reduce maternal mortality ratio by 75% by 2015 [5], [6]. In this analysis, we discuss the demographic implications of achieving MDG 5 by making the linkage between female life expectancy during the reproductive ages and maternal mortality.

Currently, a great disparity exists in maternal mortality ratios (MMR) between developed and developing countries, much larger than for any other health statistic [7], [8]. This reflects, to a large degree, differential quality in health care and health systems across countries. UN estimates for 2010 suggest that 287 thousand maternal deaths occurred worldwide, 52% of which occurred in sub-Saharan Africa [4]. The estimated maternal mortality ratio (MMR) in sub-Saharan Africa is 500 deaths per 100,000 live births, more than twice that of the global estimate of 210 deaths per 100,000 live births. The two countries with the highest maternal mortality ratios, Chad and Somalia at 1100 and 1000, respectively, are both located in this region [4].

Although high, these numbers, and the ambitious target to decrease them, are not without precedent. Strikingly high MMRs have been documented in the past in currently developed regions, such as Europe and North America. The MMR was as high as 612 per 100,000 live births in England and Wales in 1848, declining to 282 by 1939 and then to 10 by 1981 [9]. Similarly, in Sweden the MMR dropped from 1090 to 6 per 100,000 live births between 1756–60 and 1950, and from 608 to 17 per 100,000 live births between 1915 and 1953 in the U.S [9]. This pattern of rapid decline in MMRs during the first half of the 20^th^ century is common in other developed countries.

The reductions in maternal mortality are part of a greater shift in causes of mortality and morbidity that occurred in the developed world during the twentieth century, as recognized in the framework of the demographic and epidemiologic transitions [10], [11]. Today's industrialized countries have largely progressed through both transitions, reflected in the uninterrupted rise in life expectancy at birth [12], [13]. Developing countries remain at different stages of the transitions, and the contribution of maternal mortality to life expectancy estimates, particularly during reproductive-ages, remains to be studied.

The aim of this study is to evaluate the impact of maternal mortality reduction on improvement in women's life expectancy during the reproductive period (ages 15 to 49). We first establish historical understanding of the impact of reducing maternal mortality by calculating the increase in reproductive-aged life expectancy (RALE) gained by developed countries in the 20^th^ century. Secondly, we estimate what can be achieved by lowering maternal mortality in regions of the developing world that lag behind in the demographic and epidemiologic transitions, notably much of sub-Saharan Africa. In addition, as maternal mortality is just one among several competing causes of death in sub-Saharan Africa, notably HIV/AIDS, it is of interest to understand the potential gain of life expectancy by eliminating maternal mortality in the context of generalize HIV epidemic as well.

## Methods

### Ethics Statement

Because this manuscript involved secondary data analysis of public sources, ethics approval from our respective institutions, Institutional Review Board (IRB), was not required. Our IRB board specifies “Exempt research does not require continuing IRB Committee review. Examples of exempt research include surveys, or interviews without individual identification or the use of existing data, documents, or other records without individual identifiers.” [14] As described below our data did not have any individual identifiers.

### Data Sources

Two main data sources are used in this study: life tables from the Human Mortality Database (HMD) [15] and age-specific death rates from the Demographic and Health Surveys (DHS) [16]. The HMD contains detailed time series of mortality data and life tables for entire populations (without individual identification) with virtually complete registration and census data, predominantly from developed countries. Annual period life tables were extracted from this database and life expectancies between 15 and 49 were calculated from them. We supplemented this database with model-based data from Preston [17]. The latter were used to decompose the declines in mortality into its contributing causes of death, focusing on maternal mortality in developed countries. While these data may not adequately reflect mortality patterns in the individual countries, important lessons can be learned from the patterns of developed countries in the early 20^th^ century.

DHS data were used to assess the maternal mortality contribution in survival between ages 15 and 50 in sub-Saharan Africa. The DHSs are nationally-representative household surveys (without individual identification). All-cause age-specific deaths rates were used to construct country-specific life tables and estimate total potential gains in RALE. Age-specific maternal mortality rates were used to calculate the potential contributions in RALE that could be gained through elimination of maternal deaths. Maternal mortality ratios from each country were also taken from the DHS. Maternal mortality information is generally collected using either the sisterhood method or direct estimation [18], [19]. The sisterhood method asks female respondents to list all their siblings, whether each of them is alive at the time of the survey, and in the event that a sister has died, whether she was pregnant, in childbirth, or within two months of postpartum at the time of death. Direct estimation calculates MMR by collecting information on births and maternal deaths within a specified period of time, occasionally supplemented with verbal autopsy to minimize misclassification errors. Several studies have examined the quality of DHS for maternal mortality estimation and in general, agree that the sibling method is more likely to underestimate than overestimate maternal mortality rates [20], [21], [22], [23]. In general, any survey based estimations of adult mortality are slightly biased downward. As with direct estimation methods and vital registration systems, the risk of misclassification errors of maternal mortality estimates exist [24]. Finally, we supplemented the information for these countries with data on HIV prevalence rates from UNAIDS Global Report 2010 [25].

As one of the main focuses of this study is to estimate the current contribution of maternal mortality to female RALE in selected sub-Saharan African countries, we chose to include only the most recent DHS from each country and only those that were conducted between 2000 to 2011. By restricting our sample to more recent data, we also hoped to alleviate some of the measurement issues associated with maternal mortality estimation techniques applied in older DHS [20]. Other questions of data quality, such as the potential for mis- and under-reporting, exist in all the DHS, UNAIDS and Preston datasets. Though important, we felt that such data quality issues are well known and should not preclude us from conducting this analysis.

### Reproductive-Aged Life Expectancy (RALE)

We defined the term female reproductive-aged life expectancy (RALE) as female life expectancy calculated from age 15 to 49. If a life table is available, this measure can be calculated as the person-years lived between ages 15 and 49 divided by the number of survivors at age 15 (details of its calculation can be found in the Appendix S1).

If no mortality occurs between ages 15 and 49 then the average number of years lived between these ages is 35. The higher the mortality, the more the average will fall below 35. RALE is suited for studying maternal mortality impact because it is conditioned on survival to age 15 and takes into account all causes of mortality [4], [26].

The contribution of maternal mortality to RALE can be calculated using associated single decrement life table techniques, analogous to life tables that start at birth. These techniques are used to measure the hypothetical situation where only causes of death other than maternal mortality are present [27]. The new RALE from a “maternal mortality-eliminated” life table is denoted as RALE-MM. From these life tables the specific contribution of maternal mortality to the rise in the female survival between age 15 and 49 can be assessed.

## Results

### Historic trends in RALE for developed countries

**Figure 1:**
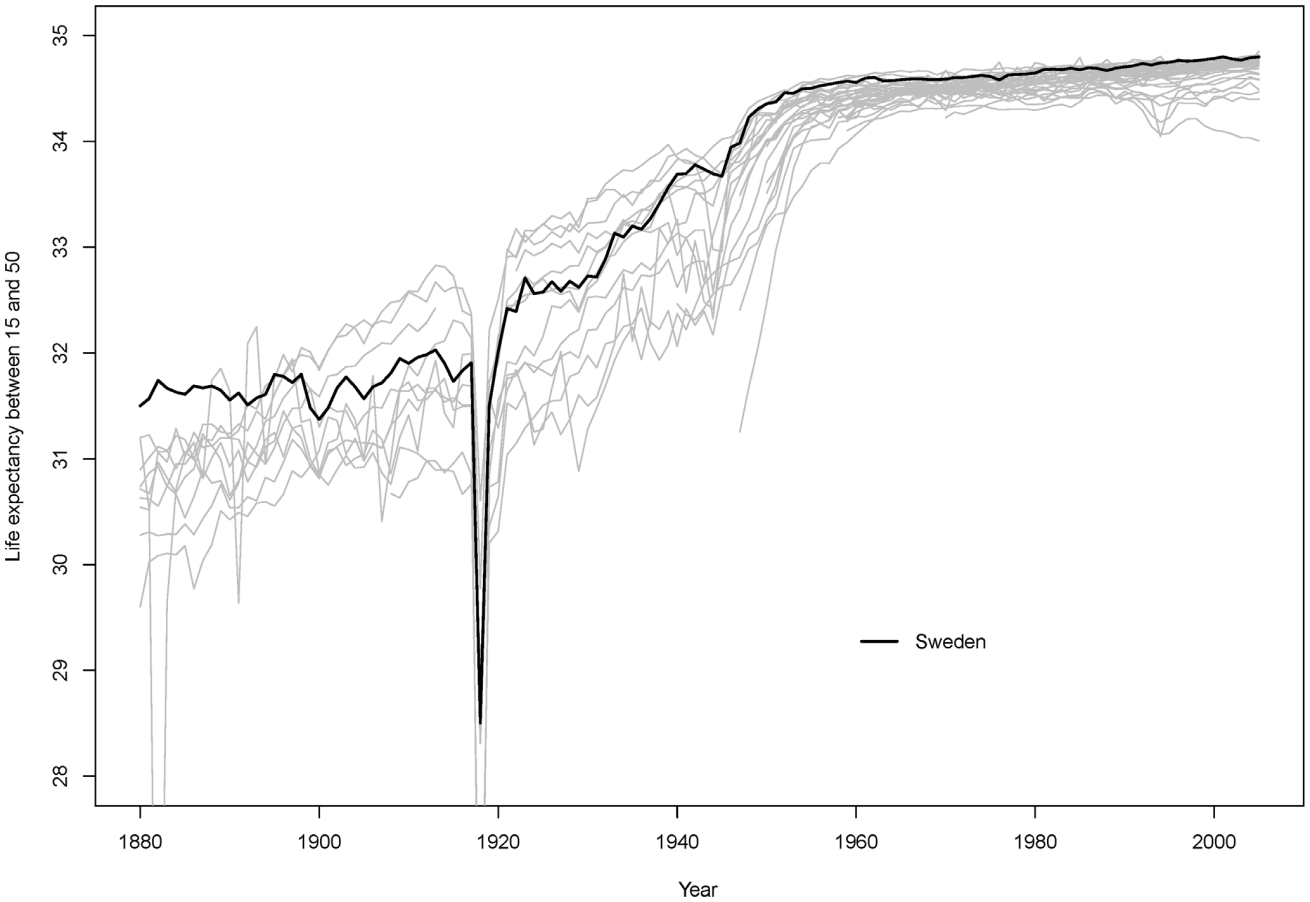
Female reproductive-aged life expectancy (RALE) from age 15 to 50 in industrialized countries, 1880–2009. Trends for Swedish females are highlighted. Source for Figure 1: Countries/regions included in the HMD: Australia, Austria, Belgium, Bulgaria, Belarus, Canada, Switzerland, Chile, Czech Republic, Germany, Denmark, United Kingdom, Spain, Estonia, Finland, France, Hungary, Ireland, Iceland, Italy, Japan, Lithuania, Luxemburg, Latvia, the Netherlands, Norway, New Zealand, Poland, Portugal, Russia, Slovak Republic, Slovenia, Sweden, Taiwan, Ukraine, and United State of America.

Figure 1 presents the country-specific trends in female RALE for developed countries with that for Sweden highlighted [15]. The relatively common pace of change in RALE for these countries and their common shift around the 1950s toward women having near-complete survival between ages 15 and 49 is profound and often unappreciated. They represent today the maximum achievable RALE for any country. As shown in the next section, maternal mortality reductions contributed to this improvement in survival.

### The Contribution of Maternal Mortality Reduction to RALE

In the industrialized world and from countries with reasonably reliable vital statistics [17] the estimated increase in RALE from 1930 to 1960 was from 29.6 to 34.5 years. Of the 4.9-year increase, half a year was gained when maternal mortality was eliminated, that is 10% of the overall gain.

A similar assessment, applied to selected countries in sub-Saharan Africa, is presented in Table 1. It shows the estimates of maternal mortality ratios by year and country, RALE with maternal mortality included and with maternal mortality eliminated (RALE-MM), and the difference between the two, i.e. the years of RALE lost to maternal mortality. The proportion of the total RALE that can be gained by eliminating maternal mortality relative to total gains in RALE expected by the elimination of all mortality is also shown. Finally, adult HIV-prevalence estimates from UNAIDS are also included in Table 1.

**Table 1:** Reproductive-aged life expectancy (RALE) in life tables with all causes of death and maternal mortality eliminated (RALE-MM), and maternal mortality ratios (MMR) and HIV prevalence rates, sub-Saharan Africa 2000–2011.

Country	Year	MMR	RALE	RALE-MM			
			35e15	35e15-MM	Difference	Proportion of total change	HIV Prevalence (15–49)
			A	B	C = B-A	C/(35-A)	
Benin	2006	397	33.2	33.6	0.4	0.23	1.2
Burundi	2010	500	32.8	33.3	0.5	0.24	3.3
Cameroon	2011	782	31.6	32.3	0.7	0.21	5.3
Chad	2004	1099	31.7	33.1	1.5	0.44	3.4
Congo	2005	781	31.6	32.2	0.7	0.19	3.4
DRC	2007	549	31.7	32.3	0.6	0.18	
Cote d'Ivoire	2005	543	31.3	31.7	0.5	0.12	3.4
Ethiopia	2011	676	32.8	33.4	0.6	0.29	
Gabon	2000	519	32.3	32.9	0.6	0.22	5.2
Ghana	2007	580	33.0	33.3	0.4	0.17	1.8
Guinea	2005	980	32.2	33.2	1.0	0.35	1.3
Kenya	2008	488	32.1	32.5	0.4	0.14	6.3
Lesotho	2009	1155	29.1	29.7	0.6	0.1	23.6
Madagascar	2008/09	498	32.8	33.3	0.5	0.21	0.2
Malawi	2010	675	30.6	31.2	0.6	0.14	11
Mali	2006	464	32.8	33.4	0.6	0.27	1
Mauritania	2000/01	747	33.3	34.0	0.7	0.38	0.7
Namibia	2006	449	30.9	31.2	0.2	0.06	13.1
Niger	2006	648	32.7	33.6	0.9	0.38	0.8
Nigeria	2008	545	32.5	33.1	0.6	0.23	3.6
Rwanda	2010	476	33.3	33.7	0.4	0.24	2.9
Senegal	2005	401	33.4	33.8	0.4	0.24	0.9
Sierra Leone	2008	857	31.7	32.6	0.9	0.26	1.6
Swaziland	2006	589	27.9	28.4	0.5	0.07	25.9
Tanzania	2010	454	32.5	33.0	0.4	0.17	5.6
Uganda	2011	438	32.2	32.7	0.5	0.18	6.5
Zambia	2007	591	28.7	29.2	0.5	0.08	13.5
Zimbabwe	2010	960	29.8	30.4	0.6	0.12	14.3

Source; based on DHS data, and author's calculations, except for HIV Prevalence (15–49) from UNAIDS Global Report 2010 (Only countries with HIV information are shown).

The RALE values range from 27.9 to 33.4 years in these sub-Saharan African populations. The possible gains by eliminating maternal mortality vary from 0.24 years in Namibia to 1.47 years in Chad, or from 6% to 44% of the total potential gain in RALE respectively.

Of the 28 countries included in this analysis, three of them, Namibia, Swaziland, and Zambia gain less than 10% of total potential RALE from the elimination of maternal mortality. Ten countries gain between 10 to 19% of total RALE and 11 countries gain between 20 and 29% from the elimination of maternal mortality. The remaining four countries of Guinea, Mauritania, Niger, and Chad would gain 35% or more of their total potential RALE gains if maternal mortality were eliminated. On average, 0.6 years of RALE can be gained by eliminating maternal mortality in sub-Saharan Africa; which is approximately 20% of total life-years gained if all mortality was eliminated between ages 15 and 49.

**Figure 2:**
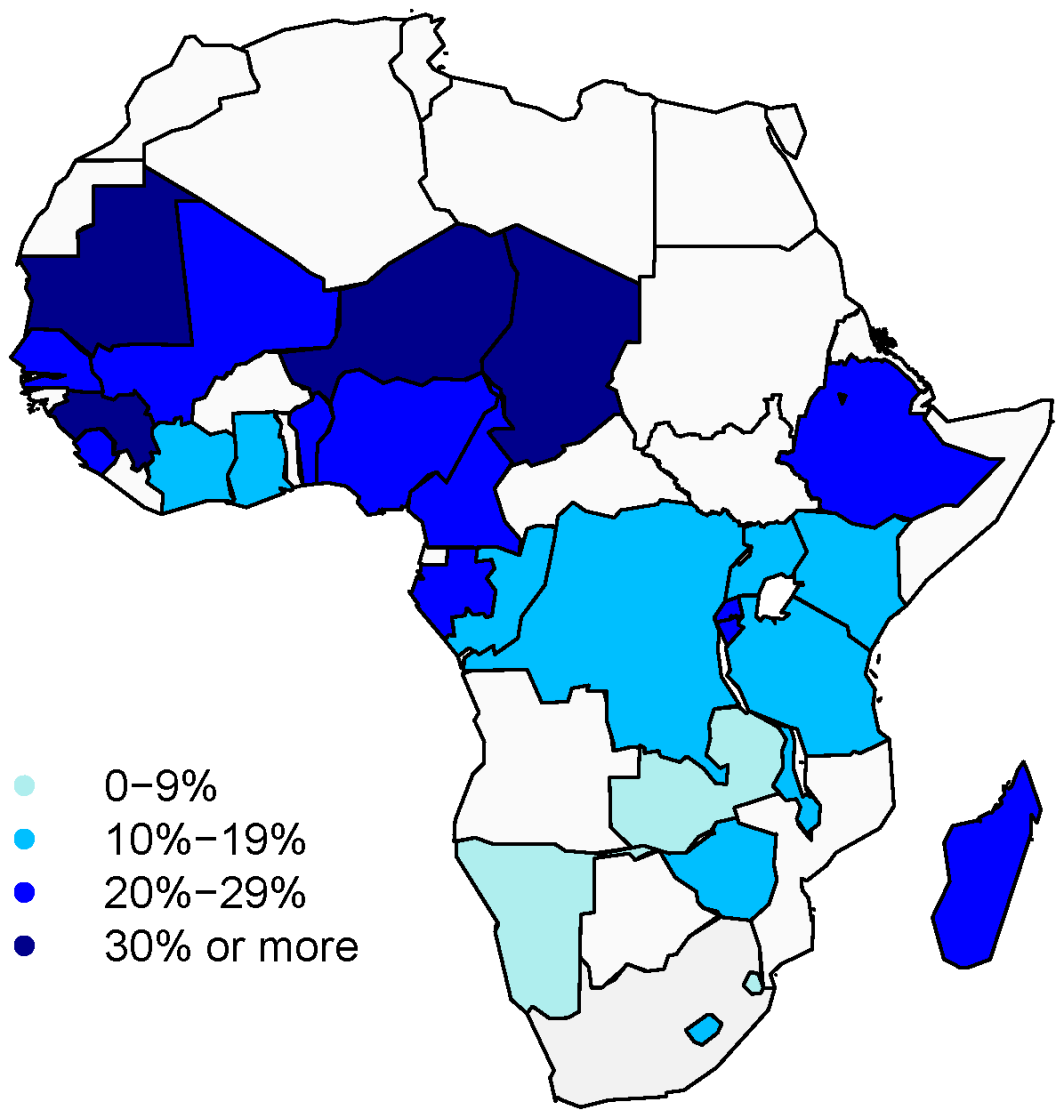
Relative proportional increase in female reproductive-aged life expectancy when eliminating maternal mortality, selected countries in Sub-Saharan Africa, 2000–2011. Source: based on DHS data, author's calculations included in the column of proportion of total change in Table 1.

**Figure 3:**
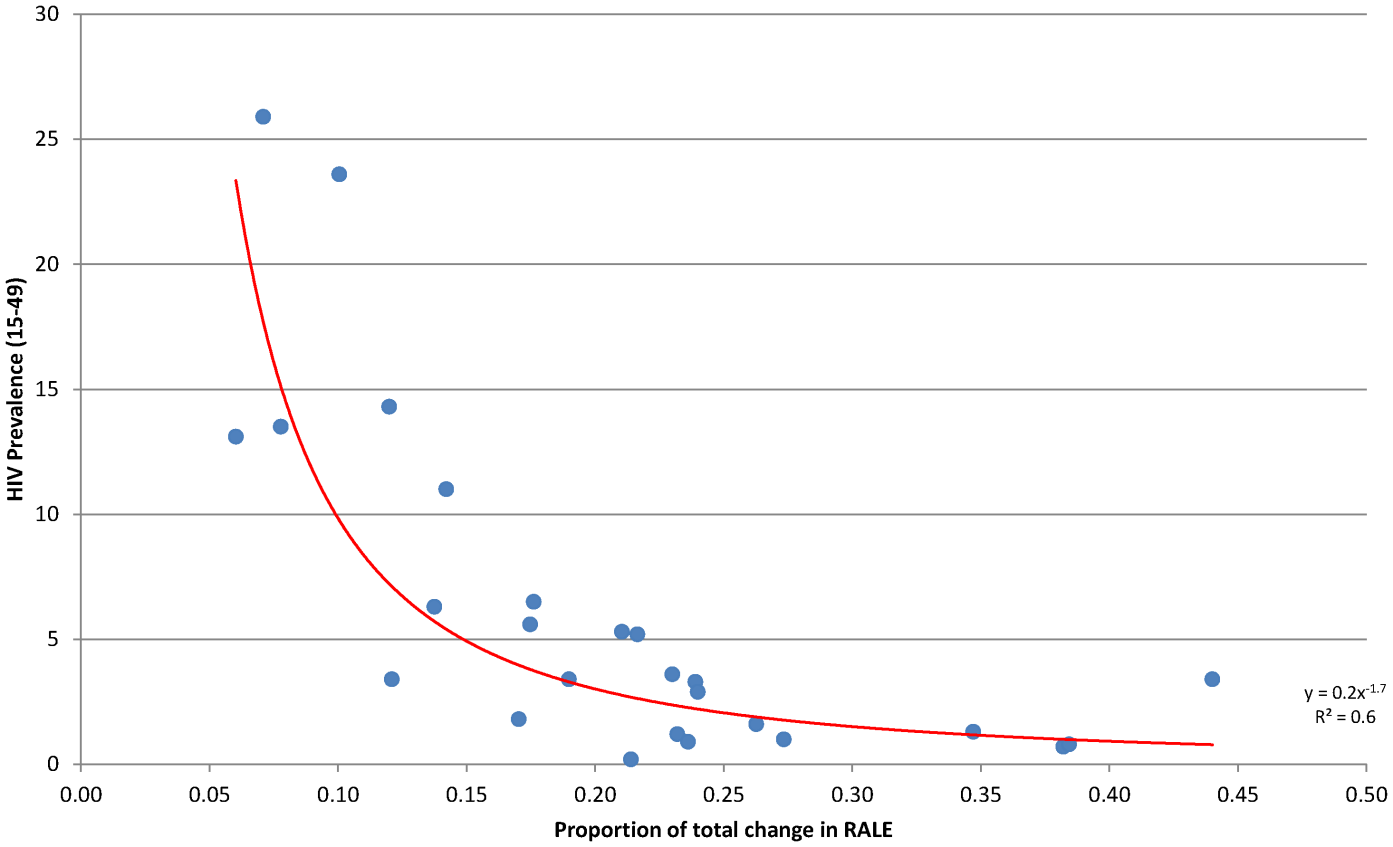
Proportion of total change in RALE gained by eliminating maternal mortality and adult HIV prevalence rate; selected African countries, 2000–2011. Source: based on DHS data, author's calculations, and UNAIDS Global Report 2010, as shown in Table 1.

When mapped in Figure 2, geographic clustering is evident, with countries in the Sahelian belt showing the highest possible relative increase in RALE if maternal mortality is eliminated. High HIV prevalence countries in the south and eastern regions, on the other hand, have the smallest possible increase. This relationship is more clearly evident in Figure 3, which plots the proportion of total gains in RALE that can be garnered by eliminating maternal mortality against the adult HIV prevalence rate as of 2005 [25]. There is a clear inverse association, suggesting that HIV is a strong competing risk of mortality during the reproductive ages for women in this region.

While constrained by the quality of the maternal mortality data from the DHS [16], we can cautiously predict that a large number of the sub-Saharan African countries will gain an average of 0.5 years in their RALE if maternal mortality is eliminated. This amount of increase mirrors the historical experience in the developed world and some other newly industrialized countries.

## Discussion and Conclusion

### Meeting the MDG 5

This analysis demonstrates the potential contribution of eliminating maternal deaths to the improvement in life expectancy between ages 15 and 49. Not previously appreciated is that, on average, a five-year gain in reproductive-aged life expectancy (RALE) for females occurred in the twentieth century in the developed and some developing countries. Of this gain, around 10% was due to the virtual elimination of maternal mortality. If maternal mortality was to be eliminated in developing countries today, the average absolute gain would be 0.6 years, higher than the 0.5 years seen in developed countries at the turn of the twentieth century. The relative percentage that this accounts for in total RALE gains is twice as high, however, 20% of potential gains in RALE in developing countries compared to 10% in developed countries. This indicates that while developing countries have begun the epidemiologic transition and other causes of death among women ages 15 to 49 have decreased, maternal mortality reductions have lagged. As a consequence, maternal mortality accounts for a larger proportion of deaths in these ages, because declines in maternal mortality have not kept pace with improved survival of other conditions.

There is wide variation in the magnitude of potential gains, however. In countries where maternal mortality makes up a high proportion of female deaths relative to other causes, the gains in life expectancy can be more than a year. Contrastingly, in places with a large HIV-burden, the potential gains in life expectancy due to the elimination of maternal mortality as a competing cause of death are comparatively low. We attempted in this analysis to estimate the relative gains in life expectancy that could be achieved by eliminating HIV. Unfortunately, the lack of age-specific AIDS-related mortality made the exercise infeasible. Figure 3 shows a clear inverse relationship between potential gains in RALE from the elimination of maternal mortality and HIV prevalence rates. It stands to reason that as the use of anti-retrovirals grows and AIDS-related mortality decreases, the proportion of deaths among women of reproductive age due to maternal mortality will likely increase.

If MDG 5 is met by 2015, female RALE can increase, on average, by 0.45 years in the 28 countries estimated here. Moreover, the contribution of reducing maternal mortality can reach as much as one year of life in some African countries. This gain in RALE may seem a small increase at a first glance, but the added survival time takes place during the most productive ages of human life and carries with it non-trivial socio-economic implications for families, workforces, and communities. Households affected by a maternal death are likely to experience reduced economic output due to the loss of mothers and wives, in addition to changes in household duties and management that may result in an increased burden on children and other household members. Infants and young children of mothers who die have been shown to have an increased risk of mortality and morbidity, increased risk of child labor at the expense of education, and reduced parental support and care [28]. The demographic implications of eliminating maternal deaths and the impact relative to competing causes of death also point to the need to better understand the distribution of causes of death, particularly HIV, and underscore the importance of gathering reliable and valid data.

The apparent ambitiousness of MDG-5 is supported by the magnitude of historical declines observed over short time frames in the Western world. More recently in some developing countries significant reductions in maternal mortality have also occurred to support the optimistic pace of change [29], [30]. Concerns have been raised about a one-size-fits-all strategy to achieve MDG-5 reductions [31]. Programmatic approaches and policies to reduce maternal mortality will need to be context specific and address other contributing factors that affect female survival through reproduction, including adequate nutrition in childhood, access to safe water and reduced exposure to malaria and HIV infection.

Maternal mortality is a relatively rare event, yet it underlies the magnitude of the RALE. In developed countries, maternal mortality statistics underestimate incidence, on average by a third, but the inaccuracies are much greater in developing countries [32], [33]. Due to probable under-reporting, DHS-based maternal mortality measurements could be under-estimated. As a result, we could have underestimated the potential gains in RALE achievable by eliminating maternal mortality. In other words, eliminating maternal mortality could lead to even greater gains in RALE in SSA. Furthermore, if information on both mortality and morbidity prevalence rates related to pregnancies and abortions were available we could study more thoroughly the health status of women over their reproductive life span [34], [35]. For example, similar to the partition of life expectancy into years lived in healthy and unhealthy states [36], RALE could be partitioned into healthy and unhealthy years.

Monitoring levels and trends in maternal mortality and causes of death in these critical ages for both women and men should be a top priority among health system strengthening efforts in all countries, and particularly in those that are lagging in their achievement of the MDG 5. If programs that help promote female education, increase access to skilled birth attendants, expand access to family planning care, and collect health information properly all continue to work together to reduce maternal mortality, the increase in RALE suggested in our results will be eventually achieved.

### Ethical statement

N/A. Because this manuscript involved secondary data analysis of a public dataset, ethics approval from our respective institution's IRB was not required.

## Supporting Information

Appendix S1 **The reproductive-aged life expectancy.**
(DOCX)
