# Supplementary material for: Potential Gains in Reproductive-Aged Life Expectancy by Eliminating Maternal Mortality: A Demographic Bonus of Achieving MDG 5
Source: PLoS One. 2014 Feb 13;9(2):e86694. doi: 10.1371/journal.pone.0086694 (PMC3923727; doi:10.1371/journal.pone.0086694)
Supplement: Appendix S1 — The reproductive-aged life expectancy. (DOCX) [file pone.0086694.s001.docx]

Appendix S1, the reproductive-aged life expectancy

In life table notation the reproductive-aged life expectancy, or RALE, can be calculated as $RALE\left( t \right)=\frac{T\left( 15,t \right)-T(50,t)}{\mathcal{l(}15,t)}$ where $T(x,t)$ and $\mathcal{l(}15,t)$ correspond to the life table measures for the person-years above age *x* and the number of survivors at age 15 at time *t*, respectively. From this equation it can be deducted that *RALE* is analogous to a life expectancy at birth from a life table that starts at age 15 and finishes at age 49, where only death rates between these ages are studied: $RALE\left( t \right)=\int_{15}^{49} e^{-\int_{15}^{a} \mu\left( x,t \right)dx}da$ , where $\mu(x,t)$ is the age-specific death rate at age *x* and time *t*.
